# Empowerment dimensions and their relationship with continuum care for maternal health in Bangladesh

**DOI:** 10.1038/s41598-021-98181-8

**Published:** 2021-09-21

**Authors:** Rushdana Rahman, Mosiur Rahman, Syed Emdadul Haque

**Affiliations:** 1 Department of Obstetrics and Gynecology, Dhaka Medical College, Dhaka, Bangladesh; 2 Department of Population Science and Human Resource Development, University of Rajshahi, Rajshahi, 6205 Bangladesh; 3 Department of Research and Training, UChicago Research Bangladesh, Dhaka, Bangladesh

**Keywords:** Health care, Public health, Epidemiology

## Abstract

One of the most important approaches to improving the health of mothers and newborns has been the continuum of care (CoC) for maternal health. Women's lack of empowerment may be an obstacle to accessing CoC in male-dominated societies. However, research often defines empowerment narrowly, despite the fact that multiple components of empowerment can play a role. The aim of this study was to look at the relationship between CoC for maternal health and measures of empowerment among Bangladeshi women. The data for this analysis came from the Bangladesh Demographic and Health Survey 2017–2018. The research centered on a subset of 4942 married women of reproductive age who had at least one live birth in the 3 years preceding the survey. Women's empowerment was measured using SWPER Global, a validated measure of women's empowerment for low- and middle-income countries. CoC for maternal health was measured at three stages of pregnancy, pregnancy, delivery, and the postpartum period. To estimate adjusted odds ratios, we specified three-level logistic regression models for our three binary response variables after descriptive analysis. Just 30.5% of mothers completed all phases of the CoC (ANC 4+, SBA, and PNC). After adjusting for individual, household, and community level variables, women with high social independence (adjusted odds ratio [AOR] 1.97; 95% confidence interval [CI] 1.58–2.47) had 97% more ANC 4+ visits, 176% higher retention in SBA (AOR 2.76; 95% CI 1.94–3.94), and 137% higher completion of full CoC (AOR 2.37; 95% CI 1.16–4.88) than women with low social independence. Frequency of reading newspapers or magazines, woman's education, age at first cohabitation, and age of the woman at first birth were significant predictors of CoC at all three stages, namely pregnancy, delivery, and postpartum, among the various indicators of social independence domain. Moreover, the intraclass correlation showed that about 16.20%, 8.49%, and 25.04%, of the total variation remained unexplained even after adjustments of individual, household and community level variables for models that predicted ANC 4+ visits, CoC from pregnancy to SBA, and CoC from delivery to the early postnatal period. The low completion rate of complete CoC for maternal health imply that women in Bangladesh are not getting the full health benefit from existing health services. Health promotion programs should target mothers with low levels of education, mothers who are not exposed to print media, and mothers who are younger at the time of birth and their first cohabitation to raise the rate of completing all levels of CoC for maternal health.

## Introduction

Although Bangladesh has made significant progress in lowering the maternal mortality ratio (MMR) from 550 per 100,000 live births in 1990 to 176 per 100,000 live births in 2015, the MMR remains remarkably high when compared to most other developing countries^1^. This country needs to reduce MMR by less than 70 per 100,000 live births by 2030 to meet the Sustainable Development Goals (SDGs) 3.1^2^. From 1990 to 2015, the average annual rate of MMR reduction in Bangladesh was only 5%^3^. Therefore, accelerating the pace of progress towards reducing maternal deaths is important in order to achieve the MMR target of SDGs in this country.

Three effective measures have been shown to minimize the MMR, including at least four visits of antenatal care (ANC), delivery by skilled birth attendant (SBA), and postnatal care (PNC) for mothers and their newborns^4,5^. However, only about 47% of pregnant women in Bangladesh obtained four or more ANC visits (ANC 4+), only 53% of births were assisted by SBA, and only 52% of women received PNC for their most recent birth within 2 days after delivery^6^. Given the importance of these three indicators, they are currently being assessed for continuity in order to ensure the survival and well-being of the mother and newborn. The continuum of care (CoC) for maternal health, which is defined as the use of continuity of health care services by women during their pregnancy, delivery, and the postpartum period^7^ is a crucial strategy for reducing maternal and newborn deaths and meeting the SDGs’ stated target.

While data on the proportion of individual maternal health care services such as ANC visits^8^, deliveries by SBA^9^, and use of PNC^10^ is available, there is no clear data on the proportion of women in Bangladesh who drop out or do not complete the CoC. Addressing each maternal service individually does not guarantee that every woman receives a comprehensive package of interventions from conception to delivery and beyond^11^. Furthermore, such a distinction obscures the fact that pregnancy and postpartum are two different cycles, each of which is shaped by the one before it^12^. Therefore, a clearer understanding of where the gaps in accessing treatment along the route are and what factors lead to the gaps is needed for successful program implementation to enhance the CoC.

Until now, there has been a large body of research on factors influencing the use of specific maternal health services^8–10,13,14^, however, there has been a dearth of research on factors influencing CoC for maternal health services. The few studies that have found that the major determinants of low rates of completion of the CoC among women in Bangladesh and other low socioeconomic settings are thought to be linked to lack of education^15^, high birth order^15^, unemployment^16^, low service accessibility^15^, unintended pregnancies^17,18^, rural residence^17^, and poor socioeconomic status^15^. However, relatively distal factors such as women's empowerment have gained little consideration in the completion of the CoC. Women's empowerment is defined as the process of empowering women with less power or influence to make informed decisions about all issues^19^ that may have an impact on the completion of the CoC.

In male-dominated societies like Bangladesh, women's autonomy in health-care decision-making is limited due to their unequal position^20^. As a result, an emphasis on women's empowerment is needed. However, the few studies done in this area^20–23^ have primarily focused on narrow definitions or limited features of empowerment, such as decision-making autonomy. While the terms autonomy and empowerment are often used interchangeably, empowerment is a multifaceted and complex concept^20,24^. We believe that the multidimensionality of women's empowerment—the fact that a woman may be highly empowered in one area, such as decision-making, but under-empowered in another, such as social independence—adds to the complexity of studies examining the links between this process and CoC completion. As a result, further empirical research is needed to obtain a better understanding of the relationship between various aspects of women's empowerment, which could contribute to progress in understanding CoC completion.

In the literature, there are a variety of indicators used to define women's empowerment^19,24–26^. One of them is the survey-based women's empowerment (SWPER Global) index, which is a useful common measure for three well-known domains of women's empowerment (attitude toward violence, social independence, and decision-making)^24^. SWPER Global is a globally standardized tool that enables to track progress over time and across countries at the individual and country levels. Therefore, in order to fill in all of the gaps in the current empirical literature, this study uses the novel, multidimensional, and validated SWPER Global index to investigate the relationships between women's empowerment and CoC completion in Bangladesh.

## Methods

### Data sources and sample

The data for this study came from the Bangladesh Demographic and Health Survey (BDHS), a nationally representative household-based survey conducted in 2017–2018^6^. A two-stage sample design was used in the 2017–2018 BDHS. In the first step, 675 primary sampling units (PSU) were created (urban areas: 250; rural areas: 425). The PSU was based on the Bangladeshi census frame from 2011. In the second stage, each PSU was given a systematic sample of 30 households on average. After removing three clusters (one urban and two rural) that were totally eroded by floodwater, the survey was successfully completed in 672 clusters. The survey comprised a total of 20,160 households. No substitutions or modifications to the pre-selected households were permitted to avoid bias^6^. The BDHS collected data from ever-married women of reproductive age (15–49 years), who spent the night before the survey in the selected households.

**Figure 1:**
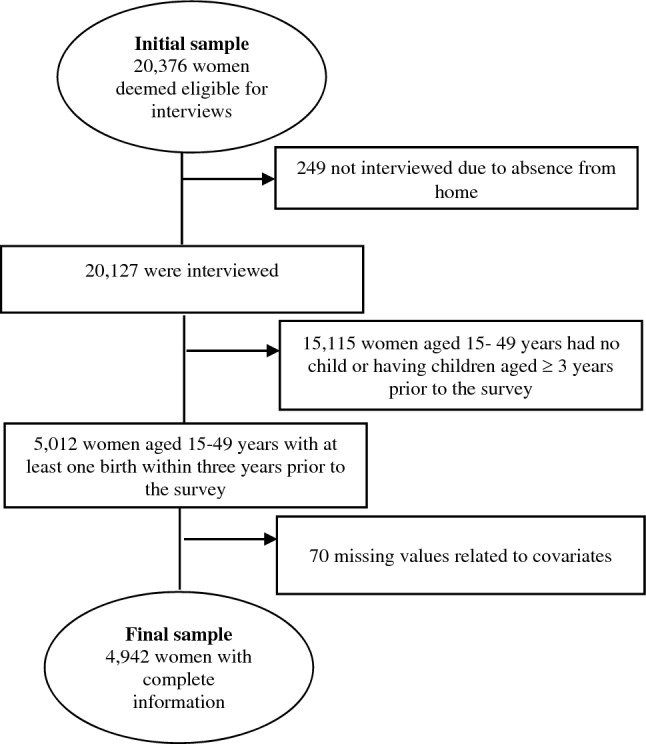
Study sample selection process: Bangladesh Demographic and Health Survey 2017–2018.

The survey used six questionnaires: (1) household; (2) women; (3) biomarker; (4) two verbal autopsies; (5) community; and (6) the fieldworker questionnaire. The questionnaires were written in English before being translated into Bangla, Bangladesh's national and official language. A pilot study was used to determine the questionnaire's reliability. Skilled data collectors conducted face-to-face interviews with an adult member of each of the 20,160 randomly selected households to obtain demographic information about the household and its members, resulting in a household response rate of 99%^6^. A survey of maternal and child health behaviors and outcomes was completed by 99% of the 20,376 women in those households who were eligible. Since data on ANC, delivery assistance, and PNC were only collected for the most recent live birth in the 3 years preceding the survey in the 2017–2018 BDHS, we enrolled 4942 women aged 15–49 years with the most recent live birth in the 3 years preceding the survey in our sample (Fig. 1).

### Outcome measures

CoC for maternal health was assessed at three levels: (1) ANC1 to ANC 4+; (2) ANC 4+ to SBA; and (3) SBA to PNC. These combined measures were dichotomized to create three binary variables (one for each outcome variable), with “1” indicating receipt of services and “0” indicating non-receipt of services. The first outcome variable contrasts women who received ANC 4+ to those who did not receive ANC 4+. As a consequence, if a woman obtained ANC 4+, the outcome was coded as “1” and “0” otherwise. The achievement of ANC 4+ visits was defined as pregnancy-level CoC. The CoC from ANC 4+ to SBA is the second outcome variable. As a result, this variable was coded "1" if a woman had ANC 4+ follow-ups and SBA assistance birth, and "0" if she had ANC 4+ but no SBA assistance birth. Achieving SBA with ANC4+ was considered to be CoC at the stage of delivery. The CoC from ANC 4+ to SBA and PNC is the third outcome variable. The outcome variable was coded “1” if a woman received ANC 4+, SBA assistance birth, and attend PNC within 48 h, and “0” if she received ANC 4+ and SBA assistance birth but did not attend PNC. The achievement of ANC4+, SBA, and PNC was assessed as continuing postpartum care, which is also defined as a complete CoC.

### Woman’s empowerment

In this study, the main independent variable of concern represents aspects of women's empowerment. Women's empowerment was measured using SWPER Global^24^, a validated measure of women's empowerment for low- and middle-income countries (LMICs). Three domains of women's empowerment were built using publicly accessible Stata programming (a Stata do-file with the original author's codes is accessible elsewhere)^24,26^: attitude toward violence, social independence, and women's decision-making power. The SWPER was created by initially employing principal component analysis to identify these three domains. Then, for each of the three domains, subindices were generated. All 14 items in the SWPER were given weights based on their loading within each domain^24^*.*

Women who read the newspaper or magazines frequently, as well as, to a lesser extent, have higher education, a later age at first birth, and smaller age and education disparities between the woman and her husband, are heavily weighted in regard to social independence. For the domain that reflected attitude to violence, women who claimed that a husband was not justified in assaulting his wife obtained a higher empowerment score than women who reported that the beating was justifiable, while all other factors were held constant. Women who said they were involved in home decisions alone or jointly also scored higher in the decision-making domain.

After creating the index, the SWPER's external validity for this expanded set of LMICs was examined through its correlation with two frequently used indices: the Gender Development Index (GDI) and the Gender Inequality Index (GII)^27^*.*

This domain of attitude towards violence, which acts as a proxy for a woman's recognition of gender norms relevant to wife-beating acceptability, includes all empowerment variables in SWPER, but is controlled by five variables that reflect women's attitude towards violence. Answers to the following questions are included: Is a husband justified in hitting or beating his wife in the following situations: (1) What happens if she goes out without informing him? (2) What if she abandons the children? (3) What if she has a disagreement with him? (4) What if she won't have sex with him? and (5) What happens if she burns the food? The following are the different types of responses: not justified = 1; justified = − 1; do not know = 0.

This domain of social independence is regarded as a prerequisite for women attaining more power. It includes all empowerment variables in SWPER but is heavily weighted by six social independence variables. These include: (1) frequency of reading the newspapers or magazines; (2) a woman's education in years of schooling completed; (3) education difference (wife's completed years of schooling minus husband's completed years of schooling); (4) gap in ages (wife's age minus husband's age); (5) age at the time of the first cohabitation; and (6) the woman's age at the time of her first childbirth.

This domain of decision-making, which measures the degree to which women participate in household decisions, includes all empowerment variables in SWPER, but is dominated by three variables that reflect women's position in household decision-making and are linked to the following questions: (1) who usually makes decisions about a respondent's health care? (2) Who makes the major purchases in the home? ((3) Who makes the decision on visiting family or relatives? The following is how the responses are coded: joint decision or respondent alone (1) or husband or other (including family elder) (− 1).

According to the SWEPER global recommendation^24^, we used standard cut points to categorize our scores into low, medium, and high levels of empowerment.

### Covariates

Relevant individual, household, and community level variables have been chosen for this analysis based on their theoretical and empirical significance in various types of literature^10–22^. Parity and pregnancy intention were included as individual level variables. Parity was determined using the tercile. To measure pregnancy intentions, a dichotomous variable was developed (intended: live birth wanted at time of conception or unintended: live birth wanted after conception or not wanted at all). The total number of household members and the household wealth index were used as household level variables in this study. Using tercile, the total number of household members was classified. Each household was allocated to the poor, middle, or rich tercile using the BDHS wealth index as a proxy indicator of household socioeconomic status. The place of residence, the distance to the nearest health facility, and transportation facilities to the sub-district headquarters were all variables at the community level. The place of residence was divided into two categories: rural and urban. The distance to the nearest health facility was determined using mothers' reports of walking hours. A variable was created to quantify transportation facilities to the sub-district headquarters that included rickshaw/van, motorcycle, car/taxi/tempo, cng/baby taxi, auto/easy bike, and others (motor lunch, animal cart, bicycle, boat, train etc.).

### Statistical analyses

STATA version 14.1 software was used to extract, clean, recode, and analyze the data. Weighting was utilized in the analysis to account for unequal probabilities of selection due to the BDHS data sampling strategy. This study measured descriptive statistics for the background characteristics of the sample as well as indicators of the SWPER empowerment index. Along the continuum, descriptive data for the rate of utilization of key maternal services were also provided. Percentages of retention and drop-offs between successive components in the CoC pathway were calculated for each of the services.

To account for clustering of observations at several levels, data were analyzed using logistic multilevel modeling approaches, the strengths and usefulness of which are extensively described^28,29^. We used a three-level binary logistic regression model to evaluate the effect of individual, household, and community-level variables on each of the three binary response outcome variables. To identify the best fitted model for each of the three outcome variables, we first created five models and compared them with Deviance, as is standard practice in multilevel analysis. These were the following: the null-model; a model with no independent variable, model I; a model adjusted solely by individual level variables, model II; a model adjusted solely by household level variables, model III; a model adjusted solely by community level variables, and model IV; a model which included individual, household, and community level variables.

Adjusted odds ratio (AOR) with 95% confidence intervals (CIs) were used to report fixed effects. The community variance, intraclass correlation coefficient (ICC), and proportional change in variance (PCV) were used to report random effects. The − 2 log-likelihood (− 2LL) statistic and Akaike Information Criteria (AIC) were used to assess the model's goodness of fit, with the lowest − 2LL and AIC indicating a better fit. The variance inflation factor (VIF) was used to examine for multicollinearity among explanatory variables. In our analysis, the VIF was < 2.0, indicating that there was no multi-collinearity issue^30^. *P* < 0.05 (2-tailed) was considered statistically significant in all tests.

### Ethical considerations

The ICF’s Independent Review Board (IRB) and Bangladesh's National Ethics Committee both approved the BDHS data collection procedures. Each respondent gave their informed consent after hearing about the survey's goals. Informed consent was obtained from a parent or legal guardian for women under the age of 18. The consent form stated explicitly the study's intent, the confidentiality of the interviews, and the respondents' rights to participate freely and withdraw from the study at any time without penalty. Since it was based on an anonymous public use of a secondary data set with no identifying information on the survey participants, this study was considered excluded from full review. All the study procedures were conducted in accordance with the principles of the Declaration of Helsinki as revised in 2013.

## Results

### Descriptive statistics

Table 1 displays the individual, household, and community level characteristics for women who gave birth in the 3 years prior to the study. There was a total of 4942 women considered. Around three-quarters of the women (73.3%) lived in rural areas and 20.9% of the births were unintended. The percentage of grand multiparous women was 29.1%. The average walking distance to the nearest health facility was 78.3 min and the most prevalent modes of transportation used to get to the sub-district headquarters were cng/baby taxis. In terms of the distribution of women's household wealth index, 67% of women were in the lowest two wealth terciles.

**Table 1:** Descriptive of individual, household, and community level characteristics of women who had a birth in the past 3 years preceding the survey: Bangladesh Demography and Health Survey 2017–2018 (n = 4942).

Characteristics	Category	Number (N)^a^	Percentage (%)^b^
**Individual level**			
**Indicators of attitude to violence**			
Beating justified if wife goes out without telling	Justified	301	6.0
	Not Justified	4639	93.9
	Do not know	2	0.04
Beating justified if wife neglects the children	Justified	454	9.2
	Not Justified	4486	90.8
	Do not know	2	0.04
Beating justified if wife argues with husband	Justified	616	12.5
	Not Justified	4322	87.6
	Do not know	4	0.08
Beating justified if wife refuses to have sex	Justified	128	2.6
	Not Justified	4804	97.2
	Do not know	10	0.2
Beating justified if wife burns the food	Justified	48	0.97
	Not Justified	4891	98.9
	Do not know	3	0.06
**Indicators of social independence**			
Frequency of reading newspapers or magazines	Not et al	4393	90.0
	< Once a week	376	7.0
	≥ Once a week	173	3.0
Woman’s education (mean ± SD)	–	–	6.9 ± 3.7
Education difference (mean ± SD)^c^	–	–	0.5 ± 3.4
Age difference (mean ± SD)^d^	–	–	− 7.9 ± 4.7
Age at first cohabitation (mean ± SD)	–	–	16.4 ± 2.8
Age at first birth (mean ± SD)	–	–	18.5 ± 3.2
**Indicators of decision making**			
Decides on respondent's health care	Joint or alone	3608	72.8
	Husband or other	1334	27.2
Decides on large household purchases	Joint or alone	3309	66.7
	Husband or other	1833	33.3
Decides on visits to family or relatives	Joint or alone	3468	69.8
	Husband or other	1474	30.2
Parity (tercile)	1	1871	37.9
	2	1623	33.0
	≥ 3	1448	29.1
Pregnancy intention^e^	Unintended	1045	20.9
	Intended	3897	79.1
**Household level**			
No. of household member (tercile)	2–4	1495	31.3
	5–6	1805	36.1
	≥ 7	1642	32.6
Household wealth index	Poor	1683	33.3
	Middle	1577	33.4
	Rich	1682	33.3
**Community level**			
Place of residence	Rural	3245	73.3
	Urban	1697	26.7
Distance to health facility, (mean ± SD)	–	–	78.3 ± 54.3
Transport facility to sub-district headquarters	Rickshaw/van	587	11.4
	Motorcycle	135	2.3
	Car/taxi/tempo	668	15.8
	CNG/baby taxi	1725	34.4
	Auto/easy bike	1628	31.9
	Others	199	4.2

^a^Number are unweighted.

^b^Percentages are weighted.

^c^Woman’s education minus husband’s education.

^d^Woman’s age minus husband’s age.

^e^Intended: live birth wanted at time of conception or unintended: live birth wanted after conception or not wanted at all.

Among the different measures of attitudes toward violence, the highest percentage accepted that beating a wife who argues with her husband was justified (12.5%; Table 1). Nine out of ten women in the study sample do not read any newspapers or magazines at all. The education distribution of the participants revealed a mean (± SD) of 6.9 (± 3.7) years of schooling. The average difference in education and age between women and their husbands was 0.5 and − 7.9 years, respectively. About 27.2%, 33.3%, and 30.2% of women had no decision-making power over their own health care, major household purchases, or visits to family or relatives respectively (Table 1).

Table 2 indicates the percent of women who received various forms of maternal health services for their most recent birth. About 47% of women earned the recommended four or more ANC visit. SBA assisted delivery accounted for 53% of all deliveries. Within the first 48 h after birth, almost half of all women and their newborns visit a PNC. Table 2 also indicates the percentages of women who received different combinations of maternal health services over the course of their care. These combinations help to highlight the components of the CoC that are closely aligned. According to the findings, only 30.5% completed all phases of the CoC (ANC 4+, SBA, and PNC).

**Table 2:** Percent distribution of women who had a birth in the past 3 years preceding the survey by different types of maternal health services received for the most recent birth: Bangladesh Demography and Health Survey 2017–2018 (n = 4942).

SN	ANC 4+^a^	SBA^b^	PNC^c^	N (%)
**Continuum of maternal care was not received**				
0	No	No	No	1575 (32.4)
**Continuum of maternal care was partially received**				
1	Yes	No	No	716 (14.5)
2	No	Yes	No	101 (2.1)
3	No	No	Yes	5 (0.1)
4	Yes	Yes	No	100 (2)
5	Yes	No	Yes	4 (0.08)
6	No	Yes	Yes	874 (18.3)
**Continuum of maternal care was received completely**				
7	Yes	Yes	Yes	1567 (30.5)
Total	47.2%	53%	49%	4942 (100%)

*Yes* received the service, *no* did not receive the service.

^a^ANC 4+ = Four or more ANC visit.

^b^SBA = Skilled birth attendant such as qualified doctors, nurses, midwives, or paramedics, family welfare visitors (FWVs), community skilled birth attendants (CSBAs), and sub-assistant community medical officers (SACMOs) at delivery.

^c^PNC = Postnatal check-up for the mother within 48 h after birth.

**Figure 2:**
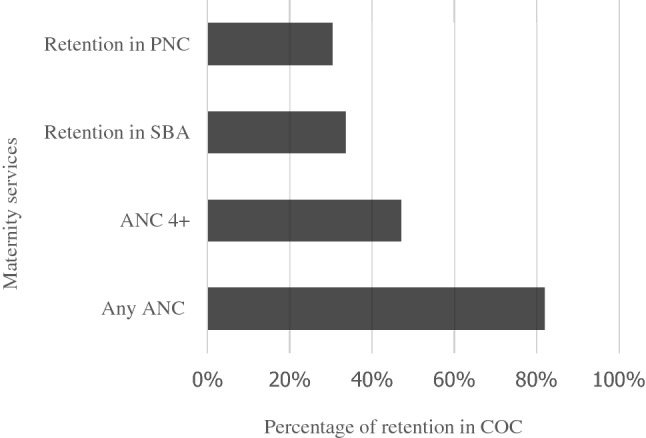
Maternal service utilization along the continuum of maternal care pathway from ANC to SBA and PNC: Bangladesh Demographic and Health Survey 2017–2018.

Figure 2 depicts service movement across the CoC to show the proportions of women who move from one maternal service to the next, as well as the points along the continuum where they terminate. Approximately 82% of the women in the total sample received ANC services at least once during their pregnancy from a medically trained provider, but a significant portion (52.8%) did not continue on the pathway to receive four or more ANC visits, the highest drop-off in the continuum. Between ANC 4+ and assisted delivery by SBA, there was a 19.1% drop-off across the continuum. Furthermore, obtaining PNC had the smallest relative drop-off (3.2%) after four or more ANC visits and assisted birth by SBA.

### Multivariate analyses

#### Random effects (measures of variation)

Measures of variation for the binary and three-level outcomes are listed in Table 3. According to the random-effects models, the full model (Model IV) revealed significant variances across the clusters in ANC 4+ visits in women who had at least one ANC and CoC from pregnancy to SBA in women who had received four or more ANC visits.

**Table 3:** Result from a random intercept model (a measure of variation) of CoC for maternal health: Bangladesh Demography and Health Survey 2017–2018 (n = 4942).

Random effects	Model 0	Model I	Model II	Model III	Model IV
**ANC 4+ (n = 4542)**^**a**^					
Community level variance (95% CI)	0.89 (0.71–1.13)	0.82 (0.64–1.05)	0.72 (0.56–0.93)	0.71 (0.55–0.92)	0.63 (0.48–0.83)
Explained variance (PCV) (%)	Ref.	7	19.10	20.22	29.21
ICC (%)	21.37	19.98	18.0	17.84	16.20
Deviance (− 2*log-likelihood)	6,027.78	5,878.47	5,871.57	5,944.69	5,744.50
AIC	6,031.79	5,900.48	5,883.57	5962.69	5,788.50
**ANC 4+ and SBA (n = 2387)**^**b**^					
Community level variance (95% CI)	1.19 (0.84–1.68)	0.94 (0.64–1.40)	0.43 (0.23–0.79)	0.72 (0.46–1.12)	0.31 (0.13–0.68)
Explained variance (PCV) (%)	Ref.	21.01	63.87	39.50	73.95
ICC (%)	26.59	22.32	11.58	17.99	8.49
Deviance (− 2*log-likelihood)	2819.08	2662.54	2560.66	2726.82	2445.64
AIC	2823.08	2684.54	2572.66	2744.82	2489.64
**ANC 4+ and SBA, and PNC (n = 1667)**^**c**^					
Community level variance (95% CI)	1.27 (0.58–2.79)	1.25 (0.56–2.85)	1.13 (0.49–2.60)	1.14 (0.49–2.62)	1.09 (0.46–2.64)
Explained variance (PCV) (%)	Ref.	1.57	11.02	10.23	14.17
ICC (%)	27.88	27.86	25.60	25.67	25.04
Deviance (− 2*log-likelihood)	743.54	728.59	733.38	730.55	710.64
AIC	747.54	750.59	745.38	748.55	754.64

*AIC* Akaike’s Information Criterion, *ICC* intra-class correlation coefficient, *PCV* proportional change in variance, *Model 0* without independent variables (null model), *Model 1* only individual-level variables, *Model II* only household-level variables, *Model III* only community-level variables, *Model IV* individual, household, and community-level variables (full model).

^a^Analyzed the predictors of four or more ANC visits by the sample of women who took at least one ANC.

^b^Analyzed the factors associated with the CoC from pregnancy to having SBA among sample of women who received at least four ANC visits.

^c^Estimates the effects of predictors on the CoC from delivery to early post-delivery period among sample of women who first received at least four ANC and SBA (i.e., completion of the entire CoC).

Our three outcomes had ICCs of 21.37%, 26.59%, and 27.88% in the null model, respectively. In other words, variances between clusters were responsible for 21.37%, 26.59%, and 27.88% of the variation in CoC from at least one ANC to ANC 4+ visits, pregnancy to SBA, and delivery to the early postnatal period in women (between-cluster variation). Furthermore, the ICC revealed that after adjusting individual, household, and community level variables for models that predicted ANC 4+ visits, CoC from pregnancy to SBA, and CoC from delivery to the early postnatal period, about 16.20%, 8.49%, and 25.04% of total variation remained unexplained.

Table 3 further reveals that the PCV rises from 7.86%, 21.01%, 1.57% (Model I) to 29.21%, 73.95%, and 14.17% (Model IV), implying that Model IV best describes the variability of our three desired outcomes. Furthermore, Model IV has the lowest − 2LL and AIK value for all three outcomes. As a result, it was chosen as the best-fitting model.

### Associations of CoC with the dimensions of women’s empowerment along with other individual, household, and community level factors (fixed effects)

After adjusting for individual, household, and community-level characteristics for our three desired outcomes, Table 4 shows the odds ratio of the full model (Model IV) for relationships between CoC and the dimensions of women's empowerment. Because the model had a greater goodness of fit, the results from Model IV for the three outcomes are reported (i.e., lower deviance).

**Table 4:** Multilevel logistic regression analysis for the associations of CoC for maternal health with the dimensions of women’s empowerment along with other individual, household, and community-level factors: Bangladesh Demography and Health Survey 2017–2018.

Variables	Adjusted odds ratio (95% CI)^1^		
	ANC 4+ (n = 4542)^2^	ANC 4+ and SBA (n = 2387)^3^	ANC 4+ and SBA, and PNC (n = 1667)^4^
**Individual-level factors**			
The SWPER index for women’s empowerment			
**Attitude to violence (ref = low)**			
Medium	1.09 (0.72–1.66)	1.47 (0.76–2.85)	0.68 (0.12–3.92)
High	1.39 (0.95–2.04)	1.85 (1.01–3.39)^c^	0.82 (0.16–4.32)
**Social independence (ref = low)**			
Medium	1.23 (1.06–1.43)^b^	1.28 (1.02–1.60)^c^	1.26 (0.76–2.09)
High	1.97 (1.58–2.47)^a^	2.76 (1.94–3.94)^a^	2.37 (1.16–4.88)^c^
**Decision making (ref = low)**			
Medium	1.06 (0.86–1.31)	1.25 (0.91–1.73)	0.87 (0.41–1.83)
High	1.09 (0.89–1.34)	1.28 (0.94–1.73)	0.89 (0.44–1.83)
**Parity (ref = 1)**			
2	0.88 (0.75–1.03)	0.57 (0.45–0.73)^a^	1.14 (0.68–1.93)
≥ 3	0.75 (0.62–0.91)^b^	0.52 (0.39–0.70)^a^	1.27 (0.67–2.39)
**Pregnancy intention (ref = intended)**			
Unintended	0.75 (0.63–0.89)^c^	0.87 (0.66–1.13)	0.55 (0.31–0.95)^c^
**Household level factors**			
**No. of household member (ref = 2–4)**			
5–6	0.88 (0.74–1.05)	1.09 (0.84–1.42)	1.24 (0.71–2.19)
≥ 7	0.95 (0.79–1.13)	0.89 (0.68–1.16)	0.98 (0.55–1.69)
**Household wealth index (ref = poor)**			
Middle	1.49 (1.25–1.78)^a^	1.85 (1.44–2.39) ^a^	1.36 (0.72–2.54)
Rich	2.26 (1.84–2.78)^a^	5.89 (4.32–8.03) ^a^	2.04 (1.04–1.99)^c^
**Community level factors**			
Place of residence (ref = rural)			
Urban	1.33 (1.07–1.65)^b^	1.18 (0.90–1.54)	0.69 (0.39–1.24)
Distance to health facility	0.96 (0.92–0.99)^a^	0.94 (0.93–0.99)^b^	0.99 (0.98–1.00)
Transport facility to sub district headquarters (Ref = rickshaw/van)			
Motorcycle	0.73 (0.39–1.38)	0.77 (0.35–1.72)	–
Car/taxi/tempo	1.16 (0.80–1.67)	0.89 (0.58–1.38)	1.26 (0.49–3.24)
CNG/baby taxi	0.64 (0.47–0.87)^b^	0.71 (0.49–1.04)	1.42 (0.61–3.29)
Auto/easy bike	0.95 (0.70–1.29)	0.71 (0.50–1.03)	1.01 (0.46–2.23)
Others	0.62 (0.36–0.97)^c^	0.33 (0.16–0.67)^b^	0.58 (0.12–0.83)^c^

*CI* confidence interval.

^1^Full model: adjusted for individual, household, and community-level factors.

^2^Analyzed the predictors of four or more ANC visits by the sample of women who took at least one ANC.

^3^Analyzed the factors associated with the CoC from pregnancy to having SBA among sample of women who received at least four ANC visits.

^4^Estimates the effects of predictors on the CoC from delivery to early post-delivery period among sample of women who first received at least four ANC and SBA (i.e. completion of the entire CoC). Here a, b, & c indicates *p* < 0.001, *p* < 0.01, & *p* < 0.05.

Full model (Model IV) that predicted ANC 4+ visits in women who had at least one ANC shows that women with medium (adjusted odds ratio [AOR] 1.23; 95% confidence interval [CI] 1.06–1.43) or high social independence (AOR 1.97; 95% CI 1.58–2.47) had 23% and 97% more ANC 4+ visits than women with low social independence. When comparing urban residents and women from rich families to rural residents and women from poor families, having four or more ANC visits was 33% and 126% higher, respectively. ANC 4+ visits were 25% less likely in grand multiparous women (≥ 3 parity) and women who had an unwanted pregnancy, respectively, than in single parity women and women who had an intended pregnancy. The odds of receiving ANC 4+ visit drop by a factor of 0.96 as the distance to a nearby health facility increases by one unit. When women used cng/baby taxis or other modes of transportation (motor lunch, animal cart, bicycle, boat, train etc.), they were 36% and 38% less likely to have ANC 4+ visits, respectively, than when they used rickshaw/van.

Full model (Model IV) that predicted the CoC from pregnancy to SBA in women who had received four or more ANC shows that women with medium (AOR 1.28; 95% CI 1.02–1.60) or high (AOR 2.76; 95% CI 1.94–3.94) social independence had a higher likelihood of remaining in SBA than women with low social independence. Furthermore, women with high negative attitude to violence dimensions were 85% more likely to likely to be retained in SBA than women with low negative attitude to violence domains. In terms of other individual, household, and community level factors women who were in the middle (AOR 1.85; 95% CI 1.44–2.39) or rich (AOR 5.89; 95% CI 4.32–8.03) wealth bands had a positive relationship with SBA retention. Two or ≥ 3 parities women, on the other hand, are 43% and 48% less likely to be retained in SBA, respectively. If the distance to a nearest health facility increases by one unit, the odds of retention in SBA decrease by a factor of 0.94. When the form of transportation was others (motor lunch, animal cart, bicycle, boat, train etc.), women were 67% less likely to be kept in SBA than when the mode of transportation was rickshaw/van.

Full model (Model IV) that predicted the CoC from delivery to the early postnatal period in women who have had at least four rounds of ANC and SBA shows that women with high social independence had a higher likelihood of completion of CoC than women with low social independence. Women in the rich band of wealth were more likely than women in the poor band of wealth to complete the entire CoC. Women whose pregnancies were unintended and whose mode of transportation to the sub-district headquarters was other (motor lunch, animal cart, bicycle, boat, train, etc.) had a lower likelihood of completing the entire CoC than women whose pregnancies were intended and whose mode of transportation was rickshaw/van.

Supplementary Table 1 shows associations of utilization of CoC for maternal care with the various indicators of dimensions of social independence. For a one unit increase in women’s education, age at first birth, and age at first cohabitation, the odds of completing the entire CoC increases by a factor of 0.08, 0.12, and 0.13, respectively. Models also demonstrates that reading a newspaper or magazine once a week was associated to CoC at all three levels.

## Discussion

This is the first study to examine data from a national survey in Bangladesh on the relationship between the CoC for maternal health and women's empowerment, assessed by a validated SWEPER Global scale for LMICs. There are five main findings: first, after initiating ANC visits, many women fell out of the continuum route and did not have four or more ANC follow-ups, SBA or PNC within 48 h of birth. As a result, just 30.5% of women in the overall sample population completed all three main elements of CoC. Second, between the ANC 4+ and the assisted delivery by the SBA, there was a high drop-off across the continuum. Third, women with high social independence had a higher rate of CoC at (1) pregnancy level; (2) delivery level; and (3) postpartum level (complete CoC). Fourth, when other individual, household, and community level variables are considered, the rich band of wealth and the mode of transportation (motor lunch, animal cart, bicycle, boat, train, etc.) have a significant relationship with the likelihood of completing the CoC at all three stages, namely pregnancy, delivery, and postpartum. Finally, other unmeasured community factors may have an impact on CoC for maternal health among Bangladeshi married women.

Findings of a low completion rate for the complete CoC for maternal health suggest that women have not benefited as much as possible from current health services in Bangladesh. The complete CoC rate observed in this study were lower than those recorded in other countries in South Asia, including India (45%)^31^, Nepal (75%)^32^, and Pakistan (40%)^33^. The rate was lower than in other developing countries, such as Cambodia (60%)^34^, Egypt (51%)^15^, and Ethiopia (45%)^17^, respectively. The low completion rate of the complete CoC for maternal health in our sample indicates a higher risk of maternal and neonatal complications, as many women could skip proven interventions at different touch points in the continuum.

Finding also found that there was a higher drop-off across the CoC between pregnancy and delivery than between delivery and postnatal period. This finding contrasts with studies in Cambodia^34^, Nepal^35^, and Pakistan^33^ that recorded the highest dropout at delivery stage. In addition, the result was inconsistent with the analysis in Ghana^36^ and Tanzania^12^ which recorded the highest postnatal dropouts. The SBA is primarily driven by facility delivery (only 3–5 percentage points among home deliveries were by SBA during 2007–2017–2018)^6,37–39^ in Bangladesh, and in facility delivery, the PNC rate is nearly universal (97%)^6,37–39^, which could explain why there is a higher drop-off across the CoC between pregnancy and delivery than between delivery and postnatal period.

Finding also found that the majority of women who had SBA at delivery tended to receive PNC. Women are expected to be more likely to be checked within 48 h of childbirth when they have been attended by a skilled health care provider. Almost all births in our study sample, which were attended by SBAs, occurred mainly in health institutions. As a result, women who gave birth in a health institution had a greater opportunity to be exposed to health education related to PNC services at the time of delivery and thus have access to information on the forms, benefits, and availability of PNC services^40^ during their stay in the health care institution. These findings show that increasing the use of SBAs, particularly delivery in health care facilities, could lead to more use of PNC and thus improve the CoC in Bangladesh.

Among all three dimensions of women's empowerment, our analyses showed that the higher social independence was associated with women's adherence to the CoC pathway, i.e., receipt of ANC 4+, receipt of ANC 4+ and assisted delivery by SBA, and completion of complete CoC. This result suggests that the social independence has an effect on the CoC for women. This result is of vital importance to public health in countries such as Bangladesh, as gender roles exist and are followed in women's everyday lives and social and cultural norms limit women's ability to make choices. In line with our studies, two recent findings in Pakistan^33,41^ have shown a significant association between social independence and the CoC. The potential reason for this may be that, where women are socially independent, they have a greater chance of participating in social activities where they can exchange knowledge relevant to maternal health services and have the ability to justify their own health care. Overall, this can have a profound impact on maternal CoC.

Among the various indicators of social independence domain, frequency of reading newspaper or magazine, woman’s education, age at first cohabitation, and age of woman at first birth were significant predictors of CoC at all three stages, namely pregnancy, delivery and postpartum. The results of the relation between the frequency of reading newspapers or magazines and the CoC are consistent with the studies conducted in Nepal^35^, Egypt^15^, and Pakistan^33^. As a result, the findings suggest that print media can help support and raise awareness of the benefits of maternal health services. On the other hand, non-exposure to print media could imply socio-economic problems and power failures.

Our findings show that, as women's education increases, the rate of CoC also rises. This finding is consistent with the results of studies conducted in other developing countries^15,33,34^. The potential explanation may be that educated women may have greater health awareness of the benefits of accessing maternity care during pregnancy, childbirth, and postnatal periods^15^. Other potential explanations may be that education is likely to improve female autonomy and help women gain greater confidence and ability to make decisions about their own health, the distribution of resources within households, and may have a better chance of receiving written information about maternal health services.

According to the results of this study, rising age at first birth and age at first cohabitation was associated with increased rate of CoC completion at all levels. These results are consistent with previous research conducted in developing settings^33,42,43^. These results suggest that a woman's age at her first birth and at first cohabitation is a significant policy issue in CoC completion. Lower ages at first birth and first cohabitation can be associated with greater welfare dependency, lower overall wages, and less involvement in paid jobs^44^, which may serve as a barrier to service use. Because of their restricted social involvement, early marriage or early age at birth may put women at a disadvantage when it comes to obtaining knowledge about the value of maternal health care and danger signs during pregnancy and childbirth^43^. Early (teenage) marriage and teenage pregnancy are common in Bangladesh^6^, necessitating the implementation of policies that delay the age of marriage and childbearing in order to increase CoC.

According to the results of this study, the wealth index is significantly associated with CoC at all three levels. The rate of CoC is higher among women from wealthy households than those of women from poor households. This finding is consistent with other related studies conducted in various international settings^15,33,34^, which found that wealth inequality was a consistent predictor of CoC. Women from richer households may have better access to resources (such as money, vehicles, or motorcycles) and have greater exposure to accessing relevant information related to maternal and child health that can help them access maternal health care services. Furthermore, it has been proposed that women from wealthier families may have greater access to standard health care facilities (such as hospitals or clinics), which may have contributed to Bangladesh's higher CoC rate^10,13^. Transportation is known to be one of the most significant nonmonetary barriers to health care access, particularly in rural locations^45^. This study demonstrated, as in other studies^45,46^, that transportation facilities can have a negative impact on the completion of a complete CoC for maternal health care.

The analysis has some strengths. First, our research is interesting in that it measured the CoC for maternal health services at three levels (pregnancy, delivery, and postpartum period), so the findings can be used to track the improvement of the level of CoC at different stages. Second, while most studies include only one or two components of empowerment, this research examines the relationship between empowerment and CoC for maternal health using the multidimensional SWPER index. Finally, the large representative sample size with a high response rate suggests that selection bias is unlikely to have an effect on the observed findings. The BDHS's use of qualified staff and validated questionnaires is also likely to minimize measurement bias in the study.

We are aware of many shortcomings in our research. First, empowerment is a multidimensional concept with no established gold standard for measuring it. However, we constructed women's empowerment indices using SWPER Global, a validated multidimensional indicator for LMICs. Second, the empowerment indices and CoC outcomes were primarily focused on self-reported perceptions and recall, which may be skewed by social desirability and prone to misclassification. To alleviate the consequences of this limitation, we chose study women who had a live birth during the 3 years preceding the survey. Third, since the data is cross-sectional, causal inference cannot be emphasized. Women's empowerment, for example, was measured after the birth of a child. Measuring empowerment before and after childbirth may provide a better understanding of the factors that influence CoC for maternal health. Nonetheless, evidence suggests that such measures are as reliable as prospective longitudinal surveys.

Finally, our adjusted regression models did not account for all potential contributing variables or barriers to care, such as history of or current issues that could impact a woman's care-seeking behavior, nor did they account for a quality approach to measuring the level of CoC, because such data were not collected in the BDHS 2017–2018. Completing four or more ANC visits, for example, did not imply that a woman received any of the recommended ANC components. Further research should develop a measurement of CoC focusing more on the dimension of quality of care.

Despite these limitations, this study has provided useful information on maternal CoC and its relationship with women's empowerment, which could be used to design programs and policies to improve the CoC for maternal health in Bangladesh.

## Conclusions

This study reveals that the completion rate of the complete CoC for maternal health was extremely low, meaning that women in Bangladesh are not getting the full health benefit from existing health services. Across all three dimensions of women's empowerment, higher social independence were associated with women's adherence to CoC at all three stages, namely pregnancy, delivery, and postpartum. Frequency of newspapers or magazines reading, women's education, age at first cohabitation, and age at first birth were all important predictors of the CoC pathway among the numerous measures of social independence domain. Health promotion programs should target mothers with low levels of education, mothers who are not exposed to print media, and mothers who are younger at the time of birth and their first cohabitation to raise the rate of completing all levels of CoC for maternal health. The wealth index and mode of transportation to sub-district headquarters are two more major household and community level determinants that should be considered in order to improve the rate of CoC. However, future longitudinal studies are needed to examine the impact of possible mechanisms mediating the relationship between women's empowerment and CoC for maternal health.

The current findings highlight the importance of initiatives, programs, and policies aimed at increasing CoC completion rates through a range of empowerment programs, with a particular emphasis on social independence. Microcredit, economic livelihoods, conditional cash transfers (CCTs)^47^, and voucher programs^48^ are examples of economic interventions that have the potential to boost CoC completion through promoting women's empowerment. In Bangladesh, a voucher program^48^ was proven to be effective in increasing uptake of the entire CoC. In Cambodia^49^, India^50^, and Uganda^51^, voucher programs that improve women's economic empowerment were found to be successful in improving reproductive health care and improving maternity follow-up.

Government of Bangladesh can also improve the efficacy of existing laws by raising the age of first marriage, which would raise the age at which women give birth, and investing more in women's education, which would raise women's social status and increase uptake of the complete CoC. Another crucial factor that can boost CoC uptake in Bangladesh is an integrated health-care system that includes collaboration and teamwork at all levels, from the home to the hospital. In Bangladesh, this concept of a home-to-hospital CoC has been found to be effective in providing appropriate health care to women from all socioeconomic backgrounds^52^*.*

However, in Bangladesh, health professional shortages have a significant impact on the home-to-hospital CoC, particularly in rural areas^53^. Efforts to give a good package of incentives to health care providers for staying and serving rural communities, which should include an enabling environment in terms of security, physical condition of the house, and a career planning potential, are critical to the gaps in the home-to-hospital CoC.

## Supplementary Information

Supplementary Table 1.

## Data Availability

The datasets used and analyzed during the current study are available from the Measure DHs website: https://dhsprogram.com/data/available-datasets.cfm.

## References

[1] WHO, UNICEF, UNFPA, World Bank Group, and the United Nations Population Division. Trends in maternal mortality: 1990 to 2015: estimates by WHO, UNICEF, UNFPA, World Bank Group, and the United Nations Population Division. Geneva: World Health Organization, (2015).

[2] United Nations. Sustainable Development Goals (SDGs): Goal 3: Ensure healthy lives and promote well-being for all at all ages. http://www.un.org/sustainabledevelopment/health/ (United Nations Statistics Division, New York, 2021).

[3] World Health Organization, UNICEF. Trends in maternal mortality: 1990–2013: Estimates by WHO, UNICEF, UNFPA, the World Bank and the United Nations population division: Executive summary (2014).

[4] Bale JR, Stoll BJ, Lucas AO (2003). Improving Birth Outcomes: Meeting the Challenge in the Developing World.

[5] WHO (2016). Standards for Improving Quality of Maternal and Newborn Care in Health Facilities.

[6] National Institute of Population Research and Training (NIPORT), and ICF (2020). Bangladesh Demographic and Health Survey 2017–2018.

[7] Kerber KJ, de Graft-Johnson JE, Bhutta ZA, Okong P, Starrs A, Lawn JE (2007). Continuum of care for maternal, newborn, and child health: From slogan to service delivery. Lancet.

[8] Rahman A, Nisha MK, Begum T, Ahmed S, Alam N, Anwar I (2017). Trends, determinants, and inequities of 4^+^ ANC utilization in Bangladesh. J. Health Popul. Nutr..

[9] Kibria GMA, Ghosh S, Hossen S, Barsha RAA, Sharmeen A, Uddin SMI (2017). Factors affecting deliveries attended by skilled birth attendants in Bangladesh. Matern. Health Neonatol. Perinatol..

[10] Rahman MM, Haque SE, Zahan MS (2011). Factors affecting the utilization of postpartum care among young mothers in Bangladesh. Health Soc. Care Community..

[11] Bryce J, Arnold F, Blanc A, Hancioglu A, Newby H, Requejo J (2013). Measurement CWGoIC: measuring coverage in MNCH: New findings, new strategies, and recommendations for action. PLoS Med..

[12] Mohan D, LeFevre AE, George A, Mpembeni R, Bazant E, Rusibamayila N (2017). Analysis of dropout across the continuum of maternal health care in Tanzania: Findings from a cross-sectional household survey. Health Policy Plan..

[13] Rahman M, Nakamura K, Seino K, Kizuki M (2012). Intimate partner violence and use of reproductive health services among married women: Evidence from a national Bangladeshi sample. BMC Public Health.

[14] Kurniati A, Chen CM, Efendi F, Berliana SM (2018). Factors influencing Indonesian women's use of maternal health care services. Health Care Women Int..

[15] Hamed AF, Roshdy E, Sabry M (2018). Egyptian status of continuum of care for maternal, newborn, and child health: Sohag governorate as an example. Int. J. Med. Sci. Public Health..

[16] Haile D, Kondale M, Andarge E, Tunje A, Fikadu T, Boti N (2020). Level of completion along continuum of care for maternal and newborn health services and factors associated with it among women in Arba Minch Zuria woreda, Gamo zone, Southern Ethiopia: A community based cross-sectional study. PLoS ONE.

[17] Shitie A, Assefa N, Dhressa M, Dilnessa T (2020). Completion and factors associated with maternity continuum of care among mothers who gave birth in the last one year in Enemay District, Northwest Ethiopia. J. Pregnancy..

[18] Khan MN, Harris ML, Loxton D (2020). Assessing the effect of pregnancy intention at conception on the continuum of care in maternal healthcare services use in Bangladesh: Evidence from a nationally representative cross-sectional survey. PLoS ONE.

[19] Doku DT, Bhutta ZA, Neupane S (2020). Associations of women's empowerment with neonatal, infant and under-5 mortality in low- and/middle-income countries: Meta-analysis of individual participant data from 59 countries. BMJ Glob. Health..

[20] Rahman M, Nakamura K, Seino K, Kizuki M (2013). Does gender inequity increase the risk of intimate partner violence among women? Evidence from a national Bangladeshi sample. PLoS ONE.

[21] Hou X, Ma N (2013). The effect of women's decision-making power on maternal health services uptake: Evidence from Pakistan. Health Policy Plan..

[22] Asratie MH, Muche AA, Geremew AB (2020). Completion of maternity continuum of care among women in the post-partum period: Magnitude and associated factors in the northwest, Ethiopia. PLoS ONE.

[23] Oh J, Moon J, Choi JW, Kim K (2020). Factors associated with the continuum of care for maternal, newborn and child health in The Gambia: A cross-sectional study using Demographic and Health Survey 2013. BMJ Open.

[24] Ewerling F, Raj A, Victora CG, Hellwig F, Coll CV, Barros AJ (2020). SWPER Global: A survey-based women's empowerment index expanded from Africa to all low- and middle-income countries. J. Glob. Health..

[25] Akseer N, Kamali M, Bakhache N, Mirza M, Mehta S, Al-Gashm S (2018). Status and drivers of maternal, newborn, child and adolescent health in the Islamic world: A comparative analysis. Lancet.

[26] Ewerling F, Lynch JW, Victora CG, van Eerdewijk A, Tyszler M, Barros AJD (2017). The SWPER index for women's empowerment in Africa: Development and validation of an index based on survey data. Lancet Glob. Health..

[27] United Nations Development Program (2016). Human Development Report 2016.

[28] Blakely T, Subramanian SV, Oakes JM, Kaufman JS (2006). Multilevel studies. Methods in Social Epidemiology.

[29] Goldstein H (2003). Multilevel Statistical Models.

[30] Hair JF, Anderson RE, Tatham RL, Black WC (1995). Multivariate Data Analysis.

[31] Krishnamoorthy Y, Majella MG, Rajaa S (2020). Equity in coverage of maternal and newborn care in India: evidence from a nationally representative survey. Health Policy Plan..

[32] Thapa J, Budhathoki SS, Gurung R (2020). Equity and coverage in the continuum of reproductive, maternal, newborn and child health services in Nepal-projecting the estimates on death averted using the LiST Tool. Matern. Child Health J..

[33] Mallick L, Rabia Z, Christina J, Johanna U (2020). Trends and the Relationship Between Maternal Health and Empowerment in Pakistan, 2012–2018. DHS Further Analysis Reports No. 128.

[34] Wang, W., Hong, R. Completing the continuum of care for maternal and newborn health in Cambodia: Who drops out? DHS Further Analysis Reports No. 85, ICF International, Calverton, MD, USA (2013).

[35] Tamang, T.M. Factors Associated with completion of continuum of care for maternal health in Nepal. In: *Proceedings of the IUSSP XXVIII International Population Conference, Cape Town, South Africa* (2017).

[36] Yeji F, Shibanuma A, Oduro A, Debpuur C, Kikuchi K, Owusu-Agei S (2015). Continuum of care in a MNCH program in Ghana: Low completion rate and multiple obstacle factors. PLoS ONE.

[37] National Institute of Population Research and Training (NIPORT), Mitra and Associates, and ICF International (2009). Bangladesh Demographic and Health Survey 2007.

[38] National Institute of Population Research and Training (NIPORT), Mitra and Associates, and ICF International (2013). Bangladesh Demographic and Health Survey 2011.

[39] National Institute of Population Research and Training (NIPORT), Mitra and Associates, and ICF International (2016). Bangladesh Demographic and Health Survey 2014.

[40] Workineh YG, Hailu DA (2014). Factors affecting utilization of postnatal care service in Amhara region, Jabitena district, Ethiopia. Sci. J. Public Health..

[41] Hearld KR, Anderson JL, Budhwani H (2018). Examining the relationship between individual characteristics, community-level traits, multidimensional empowerment, and maternal health care utilization in the Islamic Republic of Pakistan. Matern. Child Health J..

[42] Adjei KK, Kikuchi K, Owusu-Agyei S, Enuameh Y, Shibanuma A, Ansah EK, Yasuoka J, Ghana EMBRACE Implementation Research Project Team (2019). Women's overall satisfaction with health facility delivery services in Ghana: A mixed-methods study. Trop. Med. Health..

[43] Sekine K, Carter DJ (2019). The effect of child marriage on the utilization of maternal health care in Nepal: A cross-sectional analysis of Demographic and Health Survey 2016. PLoS ONE.

[44] Boden JM, Fergusson DM, John HL (2008). Early motherhood and subsequent life outcomes. J. Child Psychol. Psychiatry..

[45] Sarma S (2009). Demand for outpatient healthcare: Empirical findings from rural India. Appl. Health Econ. Health Policy..

[46] Santosh K, Emily A, Dansereau CJL (2014). Does distance matter for institutional delivery in rural India?. Appl. Econ..

[47] Schuler SR, Hashemi S (1994). Credit programs, women's empowerment, and contraceptive use in rural Bangladesh. Stud. Family Plan..

[48] Mahmood SS, Amos M, Hoque S (2019). Does healthcare voucher provision improve utilisation in the continuum of maternal care for poor pregnant women? Experience from Bangladesh. Glob. Health Action..

[49] Bellows B, Warren C, Vonthanak S, Chhorvann C, Sokhom H, Men C (2011). Evaluation of the impact of the voucher and accreditation approach on improving reproductive behaviors and status in Cambodia. BMC Public Health.

[50] Bhat R, Mavalankar DV, Singh PV, Singh N (2009). Maternal healthcare financing: Gujarat's Chiranjeevi Scheme and its beneficiaries. J. Health Popul. Nutr..

[51] Ekirapa-Kiracho E, Waiswa P, Rahman MH, Makumbi F, Kiwanuka N, Okui O (2011). Increasing access to institutional deliveries using demand and supply side incentives: Early results from a quasi-experimental study. BMC Int. Health Hum. Rights..

[52] Edwards C, Saha S (2011). From home to hospital, a continuum of care: Making progress towards Millennium Development Goals 4 and 5 in rural Bangladesh. BJOG.

[53] Elmusharaf K, Byrne E, O'Donovan D (2015). Strategies to increase demand for maternal health services in resource-limited settings: Challenges to be addressed. BMC Public Health.

