# Supplementary material for: Empowerment dimensions and their relationship with continuum care for maternal health in Bangladesh
Source: Sci Rep. 2021 Sep 21;11:18760. doi: 10.1038/s41598-021-98181-8 (PMC8455624; doi:10.1038/s41598-021-98181-8)
Supplement: Supplementary file 1 — Supplementary Table 1. [file 41598_2021_98181_MOESM1_ESM.docx]

Supplementary Table 1. Adjusted odds ratios for the associations of CoC for maternal health with several indicators of social independence dimensions.: Bangladesh Demography and Health Survey 2017-18

| Variables | Adjusted odds ratio (95% CI) | | |
| --- | --- | --- | --- |
|  | ANC 4+  (n=4,542) *^1^* | ANC 4+ & SBA  (n=2,387) *^2^* | ANC 4+, SBA, & PNC  (n=1,667) *^3^* |
| **Social independence** | | | |
| Frequency of reading newspapers or magazines (ref=not at al)  <once a week  ≥once a week | 2.40 (1.37-2.27) *^a^*  4.16 (2.77-6.25) *^a^* | 3.10 (2.16-4.44) *^a^*  6.05 (3.25-11.27) *^b^* | 1.25 (0.67-2.33)  9.15 (1.27-66.27) *^c^* |
| Woman’s education in completed single years | 1.15 (1.13-1.17) *^a^* | 1.27 (1.23-1.31) *^a^* | 1.08 (1.02-1.14) *^c^* |
| Education difference (woman’s education minus husband’s education) | 0.96 (0.94-0.97) *^a^* | 0.95 (0.93-0.98) *^a^* | 0.95 (0.90-1.01) |
| Age difference (woman’s age minus husband’s age) | 0.99 (0.98-1.01) | 0.98 (0.96-1.00) | 1.01 (0.96-1.06) |
| Age at first cohabitation | 1.09 (1.07-1.11) *^a^* | 1.19 (1.15-1.23) *^a^* | 1.13 (1.05-1.21) *^c^* |
| Age of woman at first birth | 1.10 (1.08-1.12) *^a^* | 1.17 (1.13-1.21) *^a^* | 1.12 (1.05-1.20) *^c^* |

Note: *^1,2, 3^* Models were adjusted by parity, no. of household member, wealth index, place of residence and pregnancy intention. Here a, b, & c indicates *p*<0.001, *p*<0.01, & *p*<0.05
